# Systematic Review and Meta-Analysis of Policies and Interventions that Improve Health, Psychosocial, and Economic Outcomes for Young People Leaving the Out-of-Home Care System

**DOI:** 10.1177/15248380241253041

**Published:** 2024-06-03

**Authors:** David Taylor, Bianca Albers, Georgina Mann, Jane Lewis, Russell Taylor, Philip Mendes, Geraldine Macdonald, Aron Shlonsky

**Affiliations:** 1 Monash University, Caulfield East, VIC, Australia; 2 University of Zurich, Switzerland; 3 Centre for Evidence and Implementation, London, UK; 4 University of Auckland, New Zealand; 5 University of Bristol, UK

**Keywords:** child abuse, intergenerational transmission of trauma, violence exposure, alcohol and drugs

## Abstract

Young people who transition to adulthood from out-of-home care (OOHC) are more likely to experience a range of poorer outcomes relative to their same-age peers in the community. This systematic review assessed the effectiveness of policies or interventions (hereafter “interventions”) aimed at improving housing, health, education, economic, and psychosocial outcomes for youth leaving OOHC (hereafter “care leavers”). Eleven databases of published literature were reviewed along with gray literature. Eligible studies used randomized or quasi-experimental designs and assessed interventions that provided support to care leavers prior to, during, or after they left OOHC. Primary outcomes were housing and homelessness, health and well-being, education, economic and employment, criminal and delinquent behavior, and risky behavior, while secondary outcomes were supportive relationships and life skills. Where possible, results were pooled in a meta-analysis. Certainty of evidence was assessed using Grading of Recommendations Assessment, Development and Evaluation. Fourteen studies published in 27 reports were identified that examined independent living programs (ILPs) (*n* = 5), intensive support services (*n* = 2), coaching and peer support (C&PSP) (*n* = 2), transitional housing (*n* = 1), health information or coaching (*n* = 2), and extended care (*n* = 2). All but one study was conducted in the United States. Twenty small meta-analyses were undertaken encompassing ILPs and C&PSP, with two showing results that favored the intervention with certainty. The level of confidence in each meta-analysis was considered very low. A significant risk of bias was identified in each of the included studies. While some interventions showed promise, particularly extended care, the scope and strength of included evidence is insufficient to recommend any included approach.

## Background

### Young People Leaving Care are a Vulnerable Population

Youth who experience abuse and neglect by their parents or carers can be placed in out-of-home care (OOHC) in jurisdictions where such formal systems exist. Children and young people in OOHC most often come from economically disadvantaged families (Skinner et al., 2023) and non-white young people in care are often substantially overrepresented in the foster care system (Cénat et al., 2021; Cram et al., 2015; Tilbury, 2009). OOHC takes three major forms: foster care—where care services are provided by individuals not necessarily known to the recipient; kinship or kith (friendship) care—where those providing care are connected to the recipient through blood or kin ties; and group or residential care—where care is provided in settings staffed by paid carers. Youth can experience one or more of these care types while in OOHC. While the forms of OOHC are quite different, they also have similarities: children in OOHC are often the victims of childhood trauma (Garland et al., 1996; Stein et al., 2001); minimal standards of care are required; and financial and other support, if provided, ceases when youth reach a certain age (Bergström et al., 2020). The age at which young people transition from OOHC (i.e., care leavers) varies between and within some countries—for most, formal support ceases between the ages of 18 and 21 (Gypen et al., 2017). Care leavers are often ill-equipped for independent living, and the type and amount of support they receive is insufficient to prevent adverse outcomes (Nuñez et al., 2022; Petäjä et al., 2022).

How to effectively support care leavers is a policy area of considerable contemporary cross-national interest (OECD, 2022; Strahl et al., 2020; van Breda et al., 2020). Care leavers commonly experience poorer outcomes across a range of indicators relative to their counterparts in the general population, including higher rates of homelessness, unemployment, absence from the labor force, reliance on public assistance, physical and mental health problems, and contact with the criminal justice system (Crawford et al., 2018; Doyle, 2007; Dworsky & Gitlow, 2017; Fowler et al., 2017; Greeno et al., 2019; Harrison et al., 2023). These poorer outcomes may be due to pre-existing psychological and developmental problems and other challenges arising from their traumatic experiences before entering care or while in OOHC. They may also be due to deficiencies in the care and support they receive, insufficient life skills, knowledge or training, or may simply be related to the fact that, when this support is terminated, they must fend for themselves at a much earlier age than peers who often rely on their birth families for ongoing personal and material support into their mid-twenties (Donkoh et al., 2006).

### Support Available to Young People Leaving Care

Available support clusters into two broad categories: transition support programs (TSPs) and extended care policies. TSPs vary in type and mode between jurisdictions but can exist in the form of independent living programs (ILPs), coaching or peer support programs (C&PSP), intensive individualized support services, and transitional housing programs. Policies that extend the age at which care is available allow for the provision of additional funding and other support to carers to look after young people beyond the age of 18 or the payment of allowances directly to care leavers living independently. Some jurisdictions provide this option for all care leavers, while others only do so under certain conditions, e.g., if they are engaged in education or employment. These policies have been implemented in a range of settings including: the United Kingdom (England, Scotland), Denmark, Netherlands, Norway, Canada (12 provinces and territories), Australia (all 8 states and territories), New Zealand, South Africa, the United States (25 states and the District of Colombia), and Switzerland (Andersen, 2019; Mendes & Rogers, 2020; van Breda et al., 2020).

### How the Intervention Might Work

TSPs aim to provide young people with a specific set of skills that they need to build developmental assets required to succeed on their own—for example, engage in education, obtain employment and maintain housing (O’Donnell et al., 2020). They aim to do this by providing formal training, material support, or coaching and mentoring. Their focus is on enhancing life skills, boosting confidence, expanding knowledge, and fostering the ability to build and maintain relationships. These services are typically provided toward the end of young persons’ care placement, although some extend beyond 18 years of age.

Extended care policies provide young people in OOHC with the option of continuing their existing living arrangements, facilitating more time to build maturity, identify goals, and develop skills—thereby increasing the likelihood that care leavers will be ready to live independently (Strahl et al., 2020). Young people in the community often rely on their families for ongoing relationship-based and practical support into their early twenties—a period that is characterized as “emerging adulthood.” The increasing push for extended care recognizes this and seeks to break down these structural and systemic barriers by providing care leavers with additional time to build these normative material and social relationship supports (Mendes & Rogers, 2020).

### Why Is It Important to do his Review?

Over 15 years ago, Donkoh et al. (2006) conducted the first methodologically rigorous systematic review of ILP and were unable to find any studies that met their inclusion criteria. In the period since, several reviews have explored various aspects of interventions for care leavers, all of which have limitations. Some reviews limited their scope, either to particular geographies (O’Donnell et al., 2020); to interventions delivered while youth were in care (Donkoh et al., 2006; Everson-Hock et al., 2011); to ILP (Donkoh et al., 2006; Liu et al., 2019; Yelick, 2017); or to specific outcomes (Liu et al., 2019; Randolph & Thompson, 2017). Current knowledge about the effectiveness of interventions aimed at supporting care leavers may lack sufficient breadth to inform practice and policy and may also be of limited transferability to geographically or otherwise different settings than those in which studies were conducted. Other reviews have methodological limitations, such as not conducting a transparent, systematic search (Heerde et al., 2018), or not addressing the risk of bias (ROB) of included studies (Everson-Hock et al., 2011; Woodgate et al., 2017). Others did not distinguish between methodologies that are suitable for inferring causality and those that are not (Greeson et al., 2020; Häggman-Laitila et al., 2020; Liu et al., 2019; Naccarato & DeLorenzo, 2008; Yelick, 2017). A methodologically rigorous systematic review by Sundell et al. (2020) assessed the strength of the evidence for TSP. However, they combined or “lumped” diverse interventions (i.e., ILP and C&PSP) together in their meta-analyses. By conducting a methodologically rigorous review of the current best evidence of distinct interventions designed to improve outcome for care leavers, this review meets a clear need.

## Methodology

### Objectives

The objective of this review is to assess whether policies or interventions are effective at improving housing, health, education, economic, and psychosocial outcomes for care leavers (i.e., young people leaving the OOHC system relative to services as usual).

### Search Methods

Our review methods were prespecified in a peer reviewed protocol registered with the International Prospective Register of Systematic Reviews (PROSPERO; CRD42020146999) (Taylor et al., 2021). Our search strategy encompassed 11 electronic databases (Cochrane Controlled Register of Trials via Ovid, CINAHL via EBSCO, ERIC via Proquest, PsycINFO via Ovid, MEDLINE via Ovid, EMBASE via Ovid, Sociological Abstracts via Proquest, Social Services Abstracts via Proquest, SocIndex via EBSCO, NHS Economic Evaluation Database via Ovid, and Health Technology Assessment via Ovid) with no year of publication or language restrictions. Our strategy for identifying gray literature comprised searching eight clearinghouses, government agencies, and organizations known to be undertaking or consolidating research in this area (Chapin Hall at the University of Chicago, International Research Network on Transitions to Adulthood from Care, Washington State Institute for Public Policy, Australian Institute of Family Studies, California Evidence-Based Clearinghouse for Child Welfare, Social Care Online, Analysis and Policy Observatory and Gov.UK). Authors of each included study were contacted to ascertain if they were aware of any additional literature that may be relevant. Reference lists of included studies were reviewed as were studies included in nine other reviews on this topic (Everson-Hock et al., 2011; Greeson et al., 2020; Häggman-Laitila et al., 2020; Liu et al., 2019; Naccarato & DeLorenzo, 2008; Randolph & Thompson, 2017; Sundell et al., 2020; Woodgate et al., 2017; Yelick, 2017).

### Selection Criteria

The population of interest was youth aged between 16 (to capture interventions delivered prior to leaving care) and 25 (to account for follow-up) who are not living with their birth parents/birth family; are in care, defined variously as foster care, OOHC, public care, looked after, state care, government care, kinship care, or residential care; have been placed in care due to concerns related to child maltreatment; and are transitioning from care into adult living arrangements. Interventions of interest included policies or interventions that provide support and/or assistance to help youth prior to leaving care and/or as they transition and/or after they leave care, that are delivered in the community, and that support young people to transition from their country’s statutory OOHC systems into adult living. Comparators included services as usual, another intervention, no intervention, or wait-list control. Primary outcomes were housing and homelessness, health and well-being, education, employment, criminal and delinquent behavior, and risky behavior. Secondary outcomes were supportive relationships and life skills. Studies needed to be either randomized controlled trials (RCTs) or nonrandomized studies of interventions (NRSIs) that used quasi-experimental designs.

### Data Collection

**Figure 1.**
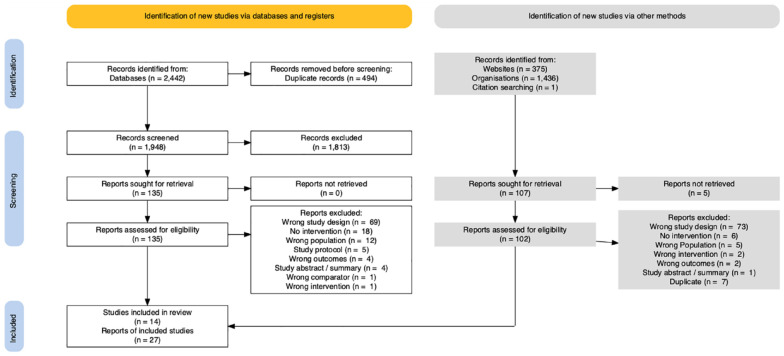
PRISMA 2020 flowchart.

Each title/abstract identified by the search strategy was independently screened by two reviewers. A third reviewer independently screened and resolved any disagreements. The full text of studies that were deemed potentially relevant at the title/abstract screening stage was further assessed by two independent reviewers against the inclusion and exclusion criteria detailed in the protocol (Taylor et al., 2021). Discrepancies and/or conflicts were resolved by discussion with an additional reviewer. Endnote was used for deduplication (The EndNote Team, 2013), Covidence was used for literature screening (Veritas Health Innovation, n.d.), and Zotero was used for library storage and referencing (Corporation for Digital Scholarship, 2022). Key steps in the identification of relevant studies are summarized in the Preferred Reporting Items for Systematic reviews and Meta-Analyses (PRISMA) 2020 flowchart (Figure 1) (Haddaway et al., 2022), a PRISMA reporting standards checklist (Rethlefsen et al., 2021) is available in the supplementary material (Taylor et al., 2024). Data extraction was undertaken by pairs of experienced reviewers, with one checking the results of the other. All reported measures that corresponded to an outcome of interest for all available time points were extracted into a data collection spreadsheet (available in the supplementary material).

### Data Analysis and Synthesis

#### Measures of Treatment Effect

Included studies reported the results of both continuous and binary measures. Standardized mean difference (SMD) was selected as the most appropriate effect size for transformation of study findings to allow us to compare the magnitude of the effect across included studies.

As Cohen’s *d* has a known bias for small studies, we used Hedges’ *g*, which corrects for this bias. For those studies that did not report sufficient data to calculate or transform effect sizes, the primary authors were contacted to request the necessary information. When information was either unavailable or insufficient to calculate an effect size, methods proposed by the Cochrane collaboration were used to transform results (Higgins et al., 2022). If this was not possible, results were presented in narrative form. Effect sizes were transformed from reported results, using the R packages *esc* and *countES* (Coxe, 2018; Lüdecke, 2019; R Core Team, 2023). We used Cohen’s (1988) benchmarks to aid in the interpretation of effect sizes by categorizing them into three levels: small (*g* = 0.2), medium (*g* = 0.5), and large (*g* = 0.8).

#### Synthesis of Results

We tabulated intervention characteristics, outcome measures, and measurement timings to assess suitability for quantitative synthesis. Meta-analyses were only conducted for outcomes that were comparable with respect to intervention type (i.e., we did not combine dissimilar interventions) and study design (i.e., we did not combine RCTs and NRSIs). For outcomes that could be quantitatively synthesized, meta-analysis was conducted using the *meta* R package (R Core Team, 2023; Balduzzi et al., 2019). A random-effects model was selected for all analyses due to the potential presence of unobserved heterogeneity within both the included studies and study populations. Outcomes that could not be synthesized quantitatively are presented narratively consistent with the Synthesis without Meta-analysis (SWiM) guidelines (Campbell et al., 2020).

#### Assessment of Bias and Confidence in Results

ROB from included RCTs was assessed using the Revised Cochrane Risk of Bias tool for randomized trials (RoB2) (Sterne et al., 2019). NRSI were assessed using the Risk of Bias in Non-randomized Studies of Interventions tool (Sterne et al., 2016). Grading of Recommendations Assessment, Development and Evaluation (GRADE) was used to summarize the confidence in meta-analyzed results (Guyatt et al., 2008). A detailed breakdown of the GRADE assessments is available in the supplementary material. Each ROB and GRADE assessment was undertaken by a pair of reviewers, with one reviewer checking the work of the other. For each meta-analysis, publication bias was assessed by producing and visually examining the symmetry of funnel plots (Borenstein et al., 2009). Egger’s test of the intercept was also used to assess funnel plot asymmetry (Egger et al., 1997).

#### Assessment of Heterogeneity

To minimize clinical heterogeneity, we carefully selected outcomes for inclusion in meta-analyses, by taking into account the characteristics of each study. Among studies that were included in a meta-analysis, consistency of results was assessed using the *I*^2^ statistic (Higgins & Thompson, 2002). Evidence of heterogeneity is highlighted in the reporting of each outcome.

#### Subgroup and Sensitivity Analysis

Insufficient studies were identified to undertake planned subgroup analysis that considered age at which statutory OOHC ends (18 vs. > 18) or gender (female vs. male). Similarly, a planned sensitivity analysis that considered study design (RCT vs. NRSI) was not possible.

### Deviations from the Protocol

Our protocol specified that we would limit our search to studies published since 1990 (Taylor et al., 2021). In practice, we did not include or exclude studies by publication date.

## Results

The electronic search strategy yielded a total of 2,442 records, of which 1,948 were unique and screened for inclusion (Figure 1). Additionally, 1,812 records from gray sources were screened. From this, 14 (*n* = 14) studies (reported in 27 papers) met our inclusion criteria and were included in this review. Studies were identified that examined ILPs (*n* = 5), intensive support services (*n* = 2), coaching and peer support (C&PSP) (*n* = 2), transitional housing (*n* = 1), health information or coaching (*n* = 2), and extended care (*n* = 2). All but one study was conducted in the United States. Eight studies used an RCT design and six used an NRSI.

Papers that reported the results of the same study or trial were treated as a single study. For these, a primary study was selected to serve as the primary reference. Papers by Kim et al. (2019) and Nadon (2020) both used data from the same source (National Youth in Transition Database). Gjertson (2016) and Blakeslee et al. (2020) undertook secondary analyses of different sets of included RCTs. To minimize bias from reporting findings from the same study twice, we included papers by Gjertson (2016), Blakeslee et al. (2020), and Nadon (2020) as secondary references of included primary studies. An overview of the included studies is provided in Table 1, with additional detail on included interventions provided in the supplementary material (Taylor et al., 2024).

**Table 1.** Summary of Included Study Characteristics.

References	Intervention and Comparison	Population (*N*)	Relevant Outcomes and Measurement Timing	Study Design and Setting
Beal et al. (2020)	*Intervention*: ICare2CHECK *Comparison*: No information	*Total*: 302 *Intervention*: 151 *Comparison*: 151 *Description*: Adolescents aged between 16 and 22 who were in child protective services for at least 12 months and expected to emancipate due to age, case plan goals and legal status	*Relevant outcomes: Health*: (a) health care use, (b) mandated appointment attendance, (c) scheduled appointment attendance *Measurement timing*: (a) baseline, (b) 12 months	*Study design*: non-randomized studies of interventions (NRSI)—Matched comparison group *Setting*: University-run medical center
Braciszewski et al. (2018)	*Intervention*: Interactive Healthy Lifestyle Preparation (iHeLP) *Comparison*: Contact control i.e., receipt of text messages	*Total*: 25 *Intervention*: 11 *Comparison*: 14 *Description*: Young people aged 18–19, who were no more than 2 years removed from foster care, who self-reported moderate or severe alcohol, tobacco or substance abuse and who received post-foster care transitions services from a large agency in New England (United States)	*Relevant outcomes: Risky behavior*: (a) substance use frequency *Measurement timing*: (a) baseline, (b) 1 month, (b) 2 months, (c) 3 months, (d) 6 months, (e) 9 months, (f) 12 months	*Study design*: randomized controlled trials (RCT) *Setting*: Delivered through participants’ smartphone
*Primary*: Courtney and Hook (2017) *Secondary*: Dworsky et al. (2013); Lee et al. (2012); Lee et al. (2014); Okpych and Courtney (2020)	*Intervention*: Extended care in Illinois (Midwest Evaluation of the Adult Functioning of Former Foster Youth) *Comparison*: Non-extended care in Wisconsin and Iowa	*Total*: 732 *Intervention*: 474 *Comparison*: 258 *Description*: Foster youth (a) who were 17 in 2002, (b) were in foster care for at least 1 year before turning 17 and (c) resided in either one of Wisconsin, Illinois and Iowa	*Relevant outcomes*: Housing and *homelessness*: (a) homelessness; *Education*: (a) high school completion or 1 or more year of college, (b) college commencement, (c) college persistence, (d) degree completion; *Criminal and delinquent behavior*: (a) arrested, (b) convicted, (c) incarcerated, (d) property crime, (e) violent crime, (f) drug crime, (g) any crime *Measurement timing*: (a) baseline, (b) 1–10 years (varies by outcome)	*Study design*: NRSI—Natural experiment (some studies use instrumental variable) *Setting*: Not applicable
*Primary*: Courtney et al. (2019); *Secondary*: Valentine et al. (2015); Skemer and Valentine (2016)	*Intervention*: YVLifeSet *Comparison*: Signposting to other resources available in the community	*Total*: 1,322 *Intervention*: 788 *Control*: 534 *Description*: Young people between 18 and 24 years of age who had been in the custody of the State of Tennessee children’s services agency for at least 1 year (not necessarily continuously) after age 14 or for at least one day after age 17.	*Housing and homelessness*: (a) homelessness, (b) couch-surfed, (c) unable to pay rent, (d) lost housing; *Health*: (a) mental health (DASS-21) *Education*: (a) high school or equivalent completion, (b) vocational training commencement, (c) college commencement; *Economic or employment*: (a) earnings, (b) employed (ever, part-time or full-time); *Criminal and delinquent behavior*: (a) robbed or assaulted, (b) incarcerated, (c) arrested, (d) convicted; *Risky behavior*: (a) days binge drinking, (b) used illegal drugs, (c) did not use condom in last sexual encounter; *Supportive relationships*: (a) social support scale, (b) very close to an adult *Measurement timing*: (a) baseline, (b) 12 months, (c) 24 months	*Study design*: RCT *Setting*: Not reported
*Primary*: Geenen et al. (2015) *Secondary*: Blakeslee et al. (2020)	*Intervention*: Better futures *Comparison*: Not reported	*Total*: 67 *Intervention*: 36 *Control*: 31 *Description*: Youth in foster care who had been identified as experiencing a significant mental health condition	*Relevant outcomes: Education*: (a) high school or equivalent completion, (b) post-secondary education commencement, (c) college commencement; *Economic or employment*: (a) employed; *Health*: (a) quality of life *Measurement timing*: (a) baseline, (b) 1 month, (c) 10 months, (d) 16 months	*Study design*: RCT *Setting*: Residential at university, coaching and workshops delivered in community
*Primary*: Greeson, Garcia, Kim, Courtney et al. (2015) *Secondary*: Courtney et al. (2011), Gjertson (2016)	*Intervention*: Massachusetts Adolescent Outreach Program for Youths in Intensive Foster Care *Comparison*: Services as usual	*Total*: 230 *Intervention*: 100 *Comparison*: 103 *Description*: Youth in foster care who have a service plan goal of independent living or long-term substitute care	*Relevant outcomes: Housing and homelessness*: (a) homelessness; Education: (a) high school or equivalent completion, (b) college commencement, (c) college persistence Economic or employment: (a) employed, (b) earnings, (c) net worth, (d) received financial assistance; *Criminal and delinquent behavior*: (a) delinquent acts; *Risky behavior*: (a) became pregnant (female), (b) got someone pregnant (male); *Life skills*: (a) overall preparedness, (b) job-related preparedness, (c) any financial accounts, (d) social security card, (e) birth certificate, (f) driver’s license; *Supportive relationships*: (a) social support *Measurement timing*: (a) baseline, (b) 12 months, (c) 24 months	*Study design*: RCT *Setting*: Not reported
*Primary*: Greeson, Garcia, Kim, Thompson et al. (2015) *Secondary*: Courtney et al. (2008), Gjertson (2016)	*Intervention*: Life Skills Training Program: Los Angeles County *Comparison*: Services as usual	*Total*: 411 *Intervention*: 196 *Comparison*: 215 *Description*: Youth aged 17 and over in out-of-home care (OOHC) in Los Angeles, California	*Relevant outcomes: Housing and homelessness*: (a) homelessness, (b) residential moves *Education*: (a) high school or equivalent completion, (b) college commencement *Economic or employment*: (a) employed, (b) earnings, (c) net worth, (d) received financial assistance; *Criminal and delinquent behavior*: (a) delinquent acts; Risky behavior: (a) became pregnant (female); *Life skills*: (a) overall preparedness, (b) job-related preparedness, (c) any financial accounts, (d) social security card, (e) birth certificate, (e) driver’s license *Measurement timing*: (a) baseline, (b) 12 months, (c) 24 months	*Study design*: RCT *Setting*: Delivered in community colleges, involved outreach in community settings
*Primary*: Kim et al. (2019) *Secondary*: Nadon (2020)	*Intervention*: Independent Living Services (National Youth in Transition Database) *Comparison*: Not reported	*Total*: 4,206 *Intervention*: 2,757 *Comparison*: 1,449 *Description*: Youth aged 17 and over (in fiscal year 2011) in foster care in the United States	*Relevant outcomes: Education*: (a) high school completion, (b) post-secondary education commencement; *Economic or employment*: (a) full-time employment *Measurement timing*: (a) baseline, (b) 4 years	*Study design*: NRSI—Propensity score matching *Setting*: Not reported
Leip (2020)	*Intervention*: True North, Broward County FL *Comparison*: Services as usual	*Total*: 326 *Intervention*: 164 *Comparison*: 162 *Description*: Youth aged 18–23 who are in foster care, extended foster care, or were involved in the foster care system, and reside in Broward County	*Relevant outcomes: Health*: (a) overall well-being; *Life Skills*: (a) job readiness; *Supportive relationships*: (a) healthy relationships *Measurement timing*: (a) baseline, (b) program exit, (c) second follow-up (7–9 months post exit).	*Study design*: RCT *Setting*: Community setting
Lim et al. (2017)	*Intervention*: New York City/New York State-Initiated Third Supportive Housing Program (NYNY III) *Comparison*: Alternative subsidized housing	*Total*: 895 *Intervention*: 251 *Comparison*: 644 *Description*: Youth aged 18–25 who are planning to leave foster care in the next 6 months or have left foster care in the last 2 years or have been in foster care for more than 1 year after their 16th birthday in New York, NY	*Relevant outcomes: Housing and homelessness*: (a) stable housing, (b) unstable housing; *Risky behavior*: (b) diagnosed sexually transmitted infection (STI) cases *Measurement timing*: (a) baseline, (b) 24 months	*Study design*: NRSI—Matching with inverse probability of treatment weighting *Setting*: Not reported
*Primary*: Powers et al. (2012) *Secondary*: Blakeslee et al. (2020)	*Intervention*: TAKE CHARGE *Comparison*: Usual Services (ILP)	*Total*: 61 *Intervention*: 29 *Comparison*: 32 *Description*: Youth who are in foster care and receive special education services	*Relevant outcomes: Education*: (a) high school or equivalent completion, (b) college commencement; *Economic or employment*: (a) employed; *Health*: (a) quality of life *Measurement timing*: (a) baseline, (b) 12 months, (c) 24 months	*Study design*: RCT *Setting*: School and community settings
Miller et al. (2020)	*Intervention*: Extended foster care in Washington *Comparison*: No extended care	*Total*: 5,715 *Intervention*: 1,751 *Comparison*: 3,948 *Description*: Youth who left foster care between 2006 and 2019 in Washington	*Relevant outcomes: Housing and homelessness*: (a) homelessness, (b) months homeless; *Health*: (a) anxiety, (b) depression, (c) any mental illness, (c) mental health treatment—outpatient or inpatient (d) diagnosed substance abuse disorder, (e) substance abuse treatment—outpatient or inpatient, (f) emergency department visits; *Economic or employment*: (a) any earnings, (b) wages; *Criminal and delinquent behavior*: (a) convictions, (b) child protection report, (c) child removed *Measurement timing*: (a) baseline, (b) 3 or 5 year follow-up (varies by outcome)	*Study design*: NRSI—Propensity score matching with inverse probability of treatment weighting *Setting*: Not applicable
Taylor et al. (2020)	*Intervention*: Premier’s Youth Initiative *Comparison*: Services as usual	*Total*: 580 *Intervention*: 290 *Comparison*: 290 *Description*: Young people leaving care (2018–2020) in target locations in New South Wales, Australia	*Relevant outcomes: Housing and homelessness*: (a) use of homelessness services *Measurement timing*: (a) baseline, (b) ~1 year	*Study design*: NRSI—propensity score matching *Setting*: Community setting
*Primary*: Zinn and Courtney (2017) *Secondary*: Courtney et al. (2011), Gjertson (2016)	*Intervention*: Independent Living—Employment Services Program, Kern County, CA *Comparison*: Did not receive intervention	*Total*: 262 *Intervention*: 140 *Comparison*: 122 *Description*: Youth who were in an OOHC placement under the guardianship of the Kern County Department of Human Services between September 2003 and July 2006 and (a) reached the age of 16 while in care or (b) entered care after age 16	*Relevant outcomes: Housing and homelessness*: (a) Homeless, (b) residential moves; *Education*: (a) high school or equivalent completion, (b) college commencement; *Economic or employment*: (a) employed, (b) earnings, (c) net worth, (d) received financial assistance; *Criminal and delinquent behavior*: (a) delinquent acts; *Risky behavior*: (a) became pregnant (female); *Life skills*: (a) overall preparedness, (b) job-related preparedness, (c) any financial accounts, (d) social security card, (e) birth certificate, (f) driver’s license *Measurement timing*: (a) baseline, (b) 12 months, (c) 24 months	*Study design*: RCT *Setting*: Community setting

### ROB Within Studies

**Figure 2.**
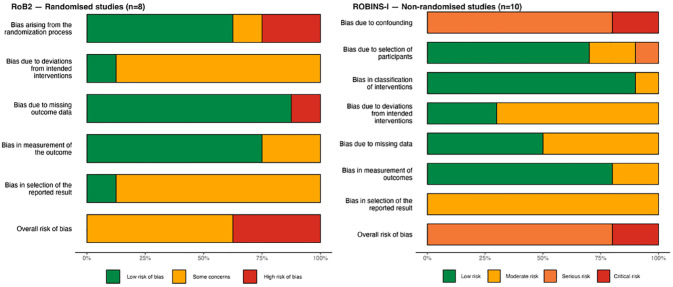
Summary of risk of bias assessments for included studies.

**Figure 3.**
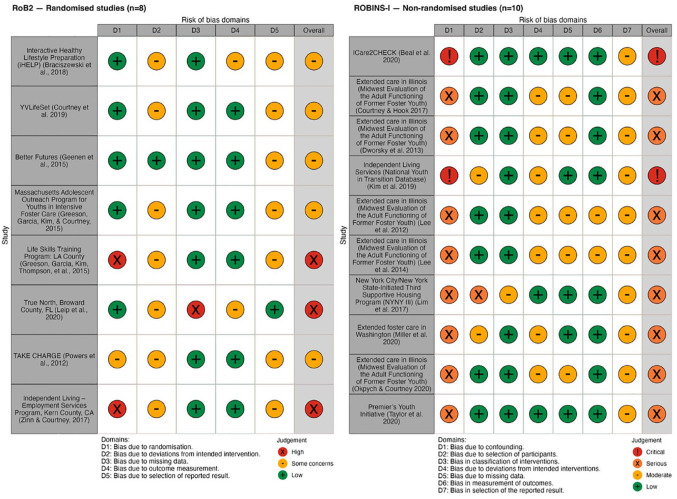
Breakdown of risk of bias assessments for included studies by domain.

Across both RCTs and NRSIs, every domain considered by each of the ROB tools was present in at least one included study (Figure 2). Additionally, each study exhibited at least one concern surrounding their ROB (Figure 3). Among included RCTs, the most common source of bias was deviations from the intended intervention, which arose from participants’ likely knowledge of their assignment to the intervention. For NRSIs, the risk of confounding was the most frequently identified issue, and the importance of this bias resulted in a decision to elevate the overall ROB to “serious” for most of these studies. Publication bias was not detected in any of the outcomes includes in meta-analyses. Detailed publication bias assessments are included in supplementary material (Taylor et al., 2024).

### Housing and Homelessness Outcomes

**Figure 4.**
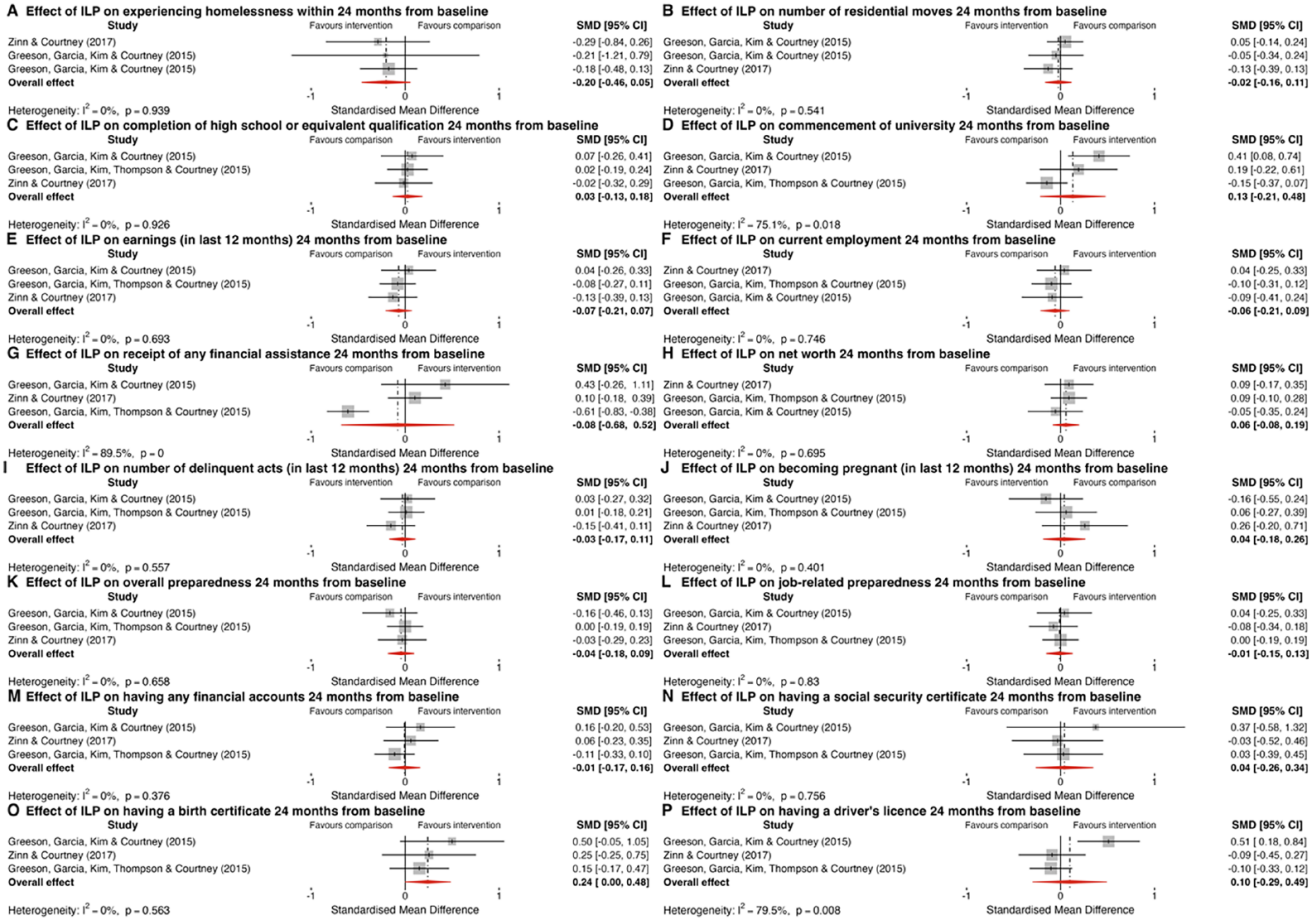
Forest plots depicting the results of meta-analysis for independent living programs.

Three RCTs assessed the impact of ILPs on participants experiencing homelessness (*I*^2^ = 0.0%, *p* = .939, Figure 4, Panel A) and on the number of residential moves they had (*I*^2^ = 0.0%, *p* = .541, Figure 4, Panel B)—a measure of housing stability—during the 2-year study period (Greeson, Garcia, Kim, Courtney et al., 2015; Greeson, Garcia, Kim, Thompson et al., 2015; Zinn & Courtney, 2017). Both outcomes were combined in separate meta-analyses. The pooled SMD for both homelessness (*g* = −0.20, 95% CI [−0.46, 0.05], *p* = .123) and number of residential moves (*g* = −0.02, 95% CI [−0.16, 0.11], *p* = .744) crossed the line of no effect, indicating that ILP had no impact on these outcomes as measured in these studies. Both meta-analyses received a very low (⊕) GRADE assessment due to being downgraded two levels for ROB (due to a high proportion of studies being assessed as having high ROB, which significantly weakens our confidence in the results) and one level for imprecision (owing to wide confidence intervals that span both clinically insignificant and potentially significant effects, indicating substantial uncertainty around the true size of the effect)—additional detail on the GRADE assessments in available in the supplementary material.

Three studies (one RCT and two NRSIs) examining the impact of TSP reported a range of relevant outcomes encompassing measures of homelessness and housing stability (Table 2). The results of the YVLifeSet RCT provide evidence of a very small effect favoring the invention in reducing homelessness, couch-surfing, and an index of housing instability; however, no effect was observed on other outcomes (Courtney et al., 2019). An NRSI of the NYNY III supportive housing intervention established that it had a medium-sized positive effect on housing stability (Lim et al., 2017). It was not possible to transform the reported result—a hazard ratio—from an NRSI of the Premier’s Youth Initiative to a common effect size, or to determine if it had a causal effect on homelessness relative to a counterfactual—as the authors only reported the results of a subgroup analysis (Taylor et al., 2020).

**Table 2.** Housing and Homelessness Outcomes Not Included in a Meta-analysis.

References	Intervention	Outcome	Outcome timing	*g* [95% CI]	Risk of Bias
Transition support programs					
Courtney et al. (2019)	YVLifeSet	Housing instability scale	12 months	0.17 [−0.29, −0.05]	Some concerns
		Experienced homelessness	12 months	−0.19 [−0.34, −0.03]	Some concerns
		Couch-surfed	12 months	−0.20 [−0.33, −0.06]	Some concerns
		Unable to pay rent	12 months	−0.11 [−0.26, 0.04]	Some concerns
		Lost housing due to inability to pay rent	12 months	−0.10 [−0.28, 0.07]	Some concerns
Lim et al. (2017)	NYNY III	Stable housing	24 months	0.51 [0.33, 0.69]	Serious concerns
Extended care policies					
Dworsky et al. (2013)	Extended care in Illinois	Homeless, age 19–21	36 months	0.57 [−1.46, 2.60]	Serious concerns
		Homeless, age 21–23	60 months	−0.14 [−0.74, 0.46]	Serious concerns
		Homeless, age 23–24	72 months	0.06 [−0.76, 0.88]	Serious concerns
Miller et al. (2020)	Extended foster care in Washington	Any homelessness, aged 18–21	36 months	−0.80 [−0.89, −0.72]	Serious concerns
		Any homelessness, age 21–23	60 months	−0.43 [−0.52, −0.34]	Serious concerns
		Average months homeless per year, aged 18–21	36 months	−0.42 [−0.48, −0.36]	Serious concerns
		Average months homeless per year, age 21–23	60 months	−0.30 [−0.37, −0.23]	Serious concerns

Two NRSIs examined the impact of extended care policies on homelessness in the United States (Table 2). An NRSI of extended care in Washington found evidence of consistent positive effects favoring the intervention on both reductions in homelessness and time spent homeless at two different follow-up points suggesting that extended care, as implemented in Washington, can have a meaningful impact on homelessness (Miller et al., 2020). Another NRSI examined the impact of extended care in Illinois at three different time points. The study found that youth who were not in extended care were more likely to be homeless when aged 19 to 21; however, this effect diminished over time, and at ages 21 to 23 and 23 to 24, there was no difference between those in extended care and those who were not. This suggests that extended care, as implemented in Illinois, had no effect on homelessness once young people had left care (Dworsky et al., 2013).

### Health and Well-being Outcomes

**Figure 5.**
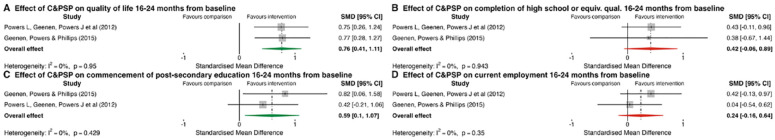
Forest plots depicting the results of meta-analysis for coaching and peer support.

Two RCTs that examined the impact of a C&PSP on quality of life (*I*^2^ = 0.0%, *p* = .950; Figure 5, Panel A) 16 to 24 months from baseline were combined in a meta-analysis. The medium-sized pooled SMD (*g* = 0.76, 95% CI [0.41, 1.11], *p* *<* .001) favors the intervention; however, this meta-analysis received a very low (⊕) GRADE assessment due to being downgraded one level for ROB and two levels for imprecision. As a result, we have very low confidence in the estimate that this intervention has an impact on quality of life as measured in this study.

Four studies (two RCTs and two NRSIs) examining the impact of TSP reported a range of health and well-being measures—with mixed results (Table 3). Positive results were reported in an RCT of YVLifeSet, which found a small reduction in depression and anxiety symptoms (Courtney et al., 2019), and an NRSI of the NYNY III supported housing intervention, which reported a large reduction in sexually transmitted infections (Lim et al., 2017). Results from an RCT of the True North TSP, measuring overall well-being (Leip, 2020) and an NRSI of ICare2Check, examining healthcare utilization (Beal et al., 2020) found no discernible difference relative to the counterfactual.

**Table 3.** Health and Well-being Outcomes Not Included in a Meta-analysis.

References	Intervention	Outcome	Outcome Timing	*g* [95% CI]	Risk of Bias
Transition support programs					
Beal et al., (2020)	ICare2CHECK	Total health care use, visits per year	12 months	1.34 [−0.51, 10.97]	Critical concerns
		Mandated foster care visits, per year	12 months	5.11 [−10.77, 111.88]	Critical concerns
		Scheduled visits, per year	12 months	3.34 [−1.17, 58.79]	Critical concerns
Courtney et al. (2019)	YVLifeSet	Mental health (DASS-21)	12 months	0.14 [0.02, 0.26]	Some concerns
Lim et al. (2017)	NYNY III	Sexually transmitted infections	24 months	−0.98 [−1.17, −0.79]	Serious concerns
Leip (2020)	True North	Overall well-being	8 months	−0.01 [−0.26, 0.23]	High
Extended care policies					
Miller et al. (2020)	Extended foster care in Washington	Anxiety, age 18–21	36 months	0.02 [−0.05, 0.1]	Serious concerns
		Depression, age 18–21	36 months	−0.02 [−0.1, 0.05]	Serious concerns
		Any mental illness, age 18–21	36 months	0.02 [−0.05, 0.1]	Serious concerns
		Mental health treatment—outpatient, age 18–21	36 months	−0.05 [−0.12, 0.03]	Serious concerns
		Mental health treatment—inpatient, age 18–21	36 months	−0.30 [−0.50, −0.10]	Serious concerns
		Diagnosed substance abuse disorder, alcohol or drug, age 18–21	36 months	−0.36 [−0.45, −0.27]	Serious concerns
		Diagnosed substance abuse disorder—alcohol, age 18–21	36 months	−0.25 [−0.37, −0.14]	Serious concerns
		Diagnosed substance abuse disorder—drug, age 18–21	36 months	−0.45 [−0.55, −0.36]	Serious concerns
		Substance abuse treatment—outpatient, age 18–21	36 months	−0.33 [−0.46, −0.19]	Serious concerns
		Substance abuse treatment—inpatient, age 18–21	36 months	−0.51 [−0.75, −0.28]	Serious concerns
		Emergency department visits, age 18–21	36 months	−0.22 [−0.28, −0.16]	Serious concerns
		Emergency department visits, age 21–23	60 months	−0.18 [−0.25, −0.12]	Serious concerns

In Washington, an NRSI of extended care examined the impact of extended care on mental health outcomes, substance use disorders, and usage of a range of health services (Table 3). Those in extended care experienced a medium-sized reduction in drug-related substance use disorders and a small reduction in alcohol-related substance use disorders. Those who received extended care used fewer health services, including inpatient and outpatient substance abuse treatment, inpatient or outpatient mental health treatment, or emergency department visits. No impact was observed on mental health outcomes (Miller et al., 2020).

### Education Outcomes

Data from three RCTs assessing the impact of ILP on high school or equivalent completion (*I*^2^ = 0.0%, *p* = .926, Figure 4, Panel C) and university commencement (*I*^2^ = 75.1%, *p* = .018; Figure 4, Panel D) within 2 years were synthesized in a meta-analysis (Greeson, Garcia, Kim, Courtney et al., 2015; Greeson, Garcia, Kim, Thompson et al., 2015; Zinn & Courtney, 2017). The overall SMD for both high school or equivalent completion (*g* = 0.03, 95% CI [−0.13, 0.18], *p* = .752) and university commencement (*g* = 0.13, 95% CI [−0.21, 0.48], *p* = .459) span the line of no effect suggesting that ILP may not have an impact on these outcomes. Both meta-analyses received a very low (⊕) GRADE assessment due to being downgraded two levels for ROB and one level for imprecision. The meta-analysis for university commencement was also downgraded one level for inconsistency (due to the evidence of substantial heterogeneity between study results, suggesting that the variation among reported effect sizes cannot be attributed solely to chance). Two RCTs that examined the impact of a C&PSP on high school graduation or GED attainment (*I*^2^ = 0.0%, *p* = .943; Figure 5, Panel B) and post-secondary education (*I*^2^ = 0.0%, *p* = .429; Figure 5, Panel C) 16–24 months from baseline were combined in separate meta-analyses (Geenen et al., 2015; Powers et al., 2012). Both meta-analyses received a very low (⊕) GRADE assessment due to being downgraded one level for ROB and two levels for imprecision. The medium-sized pooled SMD (*g* = 0.42, 95% CI [−0.06, 0.90], *p* = .085) for high school or equivalent completion favors the intervention; however, the result crosses the line of no effect, suggesting a low level of certainty that the intervention has an impact on this outcome. The pooled SMD (*g* = 0.59, 95% CI [0.10, 1.07], *p* = .018) for post-secondary education favors the intervention; however, the results of the GRADE assessment indicate that we have very low confidence that this intervention has a positive effect on this outcome.

Three studies (two RCTs and one NRSI) examined the impact of ILP on a range of education outcomes (Table 4). An RCT of YVLifeSet and an NRSI of ILPs found no impact on completion of high school or enrolment in post-secondary education (Courtney et al., 2019; Kim et al., 2019). An RCT of an ILP in Massachusetts reported a small positive effect favoring the intervention on university persistence, suggesting that it may have improved this outcome (Greeson, Garcia, Kim, Courtney et al., 2015).

**Table 4.** Education Outcomes Not Included in a Meta-analysis.

References	Intervention	Outcome	Outcome Timing	*g* [95% CI]	Risk of Bias
Transition support programs					
Greeson, Garcia, Kim, Courtney et al. (2015)	Massachusetts adolescent outreach program for youths in intensive foster care	University persistence	24 months	0.42 [0.08, 0.76]	Some concerns
Courtney et al. (2019)	YVLifeSet	Complete high school or equivalent	12 months	0.05 [−0.09, 0.20]	Some concerns
		Commence vocational training	12 months	0.18 [−0.04, 0.40]	Some concerns
		Commence post-secondary education	12 months	0.02 [−0.15, 0.19]	Some concerns
		Enrolled in 2-year college	12 months	0.1 [−0.11, 0.30]	Some concerns
		Enrolled in 4-year college	12 months	−0.03 [−0.28, 0.22]	Some concerns
Kim et al. (2019)	Independent living services	Complete high school or equivalent	48 months	0.12 [−0.02, 0.26]	Serious concerns
		Commence postsecondary education	48 months	0.10 [−0.01, 0.21]	Serious concerns
Extended care policies					
Miller et al. (2020)	Extended foster care in Washington	Complete high school or equivalent, age 18–21	36 months	0.68 [0.60, 0.77]	Serious concerns
		Undertook postsecondary education, age 18–21	36 months	0.40 [0.32, 0.49]	Serious concerns
Courtney and Hook (2017)	Extended care in Illinois	High school completion or one or more years of university, age 17–25	96 months	0.07 [0.01, 0.12]	Serious concerns
Okpych and Courtney (2020)		University enrolment, age 17–21	36 months	0.06 [0.03, 0.09]	Serious concerns
		University enrolment, 17-29/30	132 months	0.02 [−0.01, 0.04]	Serious concerns
		Two semester university persistence, age 17–21	36 months	0.04 [−0.02, 0.10]	Serious concerns
		Two-/four-year degree completion, age 17–29/30	132 months	−0.02 [−0.06, 0.01]	Serious concerns

Two NRSI examined the impact of extended care policies on education in the United States (Table 4). An NRSI of extended care in Washington reported a medium-sized positive effect on completion of high school and a small positive effect on enrolment in post-secondary education (Miller et al., 2020). Another NRSI examining the impact of extended care in Illinois found results consistent with those in Washington, reporting a very small positive effect on completing high school or commencing university by age 21. However, extended care appeared to have no effect on other measures of university enrolment, persistence, and completion (Courtney & Hook, 2017; Okpych and Courtney, 2020).

### Economic and Employment Outcomes

Three RCTs that assessed the impact of ILPs on earnings in the previous 12 months (*I*^2^ = 0.0%, *p* = .693; Figure 4, Panel E), current employment (*I*^2^ = 0.0%, *p* = .746; Figure 4, Panel F), receipt of any financial assistance (*I*^2^ = 89.6%, *p* < .001; Figure 4, Panel G), and net worth (*I*^2^ = 0.0%, *p* = .695; Figure 4, Panel H) were synthesized in four meta-analyses (Greeson, Garcia, Kim, Courtney et al., 2015; Greeson, Garcia, Kim, Thompson et al., 2015; Zinn & Courtney, 2017). The pooled results from each of the four meta-analyses—earnings in last 12 months (*g* = 0.07, 95% CI [−0.21, 0.07], *p* = .327), current employment (*g* = 0.06, 95% CI [−0.21, 0.09], *p* = .458), receipt of any financial assistance (*g* = −0.08, 95% CI [−0.68, 0.52], *p* = .796), and net worth (*g* = 0.06, 95% CI [−0.08, 0.19], *p* = .410) all indicate that ILP had no impact. All four meta-analyses received a very low (⊕) GRADE assessment due to being downgraded two levels for ROB, one level for imprecision and one level for inconsistency (financial assistance). Two RCTs examining the impact of C&PSP interventions on employment 16 to 24 months from baseline were synthesized in a meta-analysis (*I*^2^ = 0.0%, *p* = .350; Figure 5, Panel D) (Geenen et al., 2015; Powers et al., 2012). The overall SMD (*g* = 0.24, 95% CI [−0.16, 0.64], *p* = .239) suggests a small effect favoring the intervention; however, the confidence interval spans the line of no effect, suggesting a low level of certainty that there is a difference in outcomes between this intervention and services as usual. The level of certainty is further diminished by the very low (⊕) GRADE assessment, which reflects the meta-analysis being downgraded one level for ROB and two levels for imprecision.

Three studies (one RCT and two NRSIs) examined the impact of ILP on a range of employment and economic outcomes (Table 5). An RCT of YVLifeSet reported a very small positive effect on both average earnings and an index of economic hardship at 12 months; however, it appeared to have had no impact on other employment-related outcomes (Courtney et al., 2019). An NRSI examining a range of ILPs across the United States found that they had no impact on employment (Kim et al., 2019).

**Table 5.** Economic or Employment Outcomes Not Included in a Meta-analysis.

References	Intervention	Outcome	Outcome Timing	*g* [95% CI]	Risk of Bias
Transition support programs					
Courtney et al. (2019)	YVLifeSet	Average earnings	12 months	0.12 [0.00, 0.24]	Some concerns
		Ever employed	12 months	0.12 [−0.02, 0.26]	Some concerns
		Full time employment	12 months	0.01 [−0.12, 0.14]	Some concerns
		Part time employment	12 months	0.15 [−0.01, 0.32]	Some concerns
		Score on economic hardship scale	12 months	0.14 [0.02, 0.26]	Some concerns
Kim et al. (2019)	Independent Living Services	Full time employment	48 months	0.12 [−0.02, 0.26]	Critical concerns
Extended care policies					
Miller et al. (2020)	Extended foster care in Washington	Any earnings, age 18–21	36 months	0.28 [0.20, 0.35]	Serious concerns
		Any earnings, age 21–23	60 months	0.37 [0.28, 0.45]	Serious concerns
		Wages, age 18–21	36 months	0.19 [0.13, 0.25]	Serious concerns
		Wages, age 21–23	60 months	0.30 [0.23, 0.37]	Serious concerns
		Used Supplemental Nutrition Assistance Program (SNAP), age 18–21	36 months	−0.61 [−0.69, −0.54]	Serious concerns
		Used SNAP, age 21–23	60 months	−0.24 [−0.32, −0.16]	Serious concerns
		Average months used SNAP per year, age 18–21	36 months	−0.53 [−0.59, −0.46]	Serious concerns
		Average months used SNAP per year, age 21–23	60 months	−0.19 [−0.26, −0.12]	Serious concerns
		Any Temporary Assistance for Needy Families (TANF), age 18–21	36 months	−0.55 [−0.65, −0.45]	Serious concerns
		Any TANF, age 21–23	60 months	−0.51 [−0.65, −0.38]	Serious concerns
		Average months used TANF per year, age 18–21	36 months	−0.30 [−0.36, −0.24]	Serious concerns
		Average months used TANF per year, age 21–23	60 months	−0.23 [−0.30, −0.17]	Serious concerns

An NRSI of extended care in Washington examined the impact of extended care policies on earnings and use of public assistance (Table 5). The study found that extended care had a small, positive impact for both earnings and wages for those who received it, with the effect increasing over time. Those who received extended care were also much less likely to use either Supplemental Nutrition Assistance Program (SNAP) aka “food stamps” or Temporary Assistance for Needy Families (TANF). Those who received either type of assistance (SNAP or TANF) were enrolled in these programs for less time (Miller et al., 2020).

### Criminal and Delinquent Behavior Outcomes

Three RCTs assessing the impact of ILP on committing a delinquent act within the 2-year study period were combined in a meta-analysis (*I*^2^ = 0.0%, *p* = .557; Figure 4, Panel I) (Greeson, Garcia, Kim, Courtney et al., 2015; Greeson, Garcia, Kim, Thompson, et al., 2015; Zinn & Courtney, 2017). The pooled SMD (*g* = −0.03, 95% CI [−0.17, 0.11], *p* = .668) suggests that ILP has no effect on self-reported youth delinquency. This meta-analysis received a very low (⊕) GRADE assessment due to being downgraded two levels for ROB and one level for imprecision.

One RCT examined the impact of an ILP on a range of measures that examined whether participants committed violent, criminal, or delinquent activity (Table 6). The results of this RCT suggest that YVLifeSet did not have an impact on whether participants were arrested, incarcerated, or convicted 12 months from commencement. Additionally, it did not have an impact on an index of criminal behavior that included self-reported measures of criminal or delinquent behavior (Courtney et al., 2019).

**Table 6.** Criminal and Delinquent Behavior Outcomes Not Included in a Meta-analysis.

References	Intervention	Outcome	Outcome Timing	*g* [95% CI]	Risk of Bias
Transition support programs					
Courtney et al. (2019)	YVLifeSet	Arrested	12 months	−0.05 [−0.20, 0.10]	Some concerns
		Convicted	12 months	0.10 [−0.08, 0.28]	Some concerns
		Incarcerated	12 months	−0.07 [−0.22, 0.09]	Some concerns
		Criminal behavior scale	12 months	0.03 [−0.09, 0.15]	Some concerns
Extended care policies					
Miller et al. (2020)	Extended foster care in Washington	Convicted, age 18–21	36 months	−0.56 [−0.65, −0.47]	Serious concerns
		Convicted, age 21–23	60 months	−0.44 [−0.55, −0.34]	Serious concerns
Lee et al. (2012)	Extended care in Illinois (Midwest Evaluation of the Adult Functioning of Former Foster Youth)	Arrested—Female, age 17–21	48 months	−0.40 [−0.56, −0.25]	Serious concerns
		Arrested—Male, age 17–21	48 months	−0.25 [−0.45, −0.04]	Serious concerns
		Incarcerated—Female, age 17–21	48 months	−0.36 [−0.57, −0.16]	Serious concerns
		Incarcerated—Male, age 17–21	48 months	−0.19 [−0.45, 0.07]	Serious concerns
		Convicted—Female, age 17–21	48 months	−0.35 [−0.57, −0.13]	Serious concerns
		Convicted—Male, age 17–21	48 months	−0.02 [−0.39, 0.34]	Serious concerns
		Commit violent crime—Female, age 17–21	48 months	−0.03 [−0.38, 0.31]	Serious concerns
		Commit violent crime—Male, age 17–21	48 months	0.13 [−0.29, 0.55]	Serious concerns
		Commit property crime—Female, age 17–21	48 months	0.01 [−0.35, 0.37]	Serious concerns
		Commit property crime—Male, age 17–21	48 months	−0.31 [−0.52, −0.10]	Serious concerns
		Commit drug crime—Female, age 17–21	48 months	−0.19 [−0.51, 0.14]	Serious concerns
		Commit drug crime—Male, age 17–21	48 months	−0.25 [−0.49, −0.02]	Serious concerns
		Commit any crime—Female, age 17–21	48 months	0.20 [−0.24, 0.64]	Serious concerns
		Commit any crime—Male, age 17–21	48 months	0.10 [−0.27, 0.47]	Serious concerns

Two NRSIs examined the impact of extended care policies on education in the United States (Table 6). An NRSI in Washington found that extended care caused a medium-sized reduction in care leavers who were convicted of crimes between the ages of 18 to 21, and this effect persisted—albeit as a smaller one—for those aged 21 to 23 (Miller et al., 2020).

The impact of extended care in Illinois by age 21 was assessed in two reports of the same NRSI. This study used separate gender-based models to assess the impact of extended care on self-reported criminal activity, arrest, conviction, and incarceration. For women, extended care led to small reductions in arrest, incarceration, and conviction by age 21. For men, small reductions were observed in arrest and committing drug or property offenses in the same time period (Lee et al., 2012).

### Risky Behavior Outcomes

Three RCTs assessing the impact of ILP on whether a young woman became pregnant within the 2-year study period were combined in a meta-analysis (*I*^2^ = 0.0%, *p* = .401; Figure 4, Panel J) (Greeson, Garcia, Kim, Courtney et al., 2015; Greeson, Garcia, Kim, Thompson et al., 2015; Zinn & Courtney, 2017). The pooled SMD (*g* = 0.04, 95% CI [−0.18, 0.26], *p* = .731) indicates that ILP had no effect on this outcome. This meta-analysis received a very low (⊕) GRADE assessment due to being downgraded two levels for ROB and one level for imprecision.

Two RCTs examined the impact of two different TSPs on a series of risky behavior measures that focused on substance use (Table 7). The results from the RCT of YVLifeSet found no difference between intervention and comparison groups (Courtney et al., 2019). A pilot RCT of the iHeLP mobile application reported a medium-sized effect on substance abstinence favoring the intervention at 12 months; however, the estimate is underpowered, is imprecise, and crosses the line of no effect (Braciszewski et al., 2018).

**Table 7.** Risky Behavior Outcomes Not Included in a Meta-analysis.

References	Intervention	Outcome	Outcome Timing	*g* [95% CI]	Risk of Bias
Transition support programs					
Courtney et al. (2019)	YVLifeSet	Days of binge drinking in last month	12 months	−0.08 [−0.20, 0.04]	Some concerns
		Used illegal drugs (in last year)	12 months	−0.03 [−0.17, 0.11]	Some concerns
Braciszewski et al. (2018)	iHeLP	Percent days abstinent	12 months	0.37 [−0.33, 1.07]	Some concerns
Extended care policies					
Miller et al. (2020)	Extended foster care in Washington	Parented a child, age 21–23	60 months	−0.43 [−0.54, −0.32]	Serious concerns
		Child reported to child protective services, age 21–23	60 months	−0.59 [−0.98, −0.20]	Serious concerns
		Child in foster care, age 21–23	60 months	−1.23 [−2.33, −0.13]	Serious concerns

One NRSI examined the impact of extended care on a series of risky behavior outcomes related to child welfare (Table 7). The study found consistent effects favoring the intervention, suggesting that young people who received extended care in Washington were less likely to parent a child and less likely to have a child reported to child protective services or removed and placed in foster care up until age 23 (Miller et al., 2020).

### Supportive Relationships Outcomes

Two RCTs examined the impact of two different TSP on a series of measures that sought to explore supportive relationships (Table 8). All results from both studies were very small and spanned the line of no effect, indicating that neither YVLifeSet nor True North had an impact on supportive relationships as measured in these studies (Courtney et al., 2019; Leip, 2020).

**Table 8.** Supportive Relationships Outcomes Not Included in a Meta-analysis.

References	Intervention	Outcome	Outcome Timing	*g* [95% CI]	Risk of Bias
Courtney et al. (2019)	YVLifeSet	Social support scale	12 months	0.05 [−0.07, 0.17]	Some concerns
		Very close to an adult	12 months	0.05 [−0.18, 0.29]	Some concerns
Leip (2020)	True North	Healthy relationships index	8 months	0.14 [−0.10, 0.37]	High

### Life Skills Outcomes

Three studies assessing the impact of ILPs on perceived preparedness—either job-related (*I*^2^ = 0.0%, *p* = .830; Figure 4, Panel L) or overall (*I*^2^ = 0.0%, *p* = .657; Figure 4, Panel K) or over the course of the 2-year study—were synthesized in separate meta-analyses (Greeson, Garcia, Kim, Courtney et al., 2015; Greeson, Garcia, Kim, Thompson et al., 2015; Zinn & Courtney, 2017). Pooled SMDs for job-related preparedness (*g* = −0.01, 95% CI [−0.15, 0.13], *p* = .864) or overall preparedness (*g* = −0.04, 95% CI [−0.18, 0.09], *p* = .527) suggest that ILPs do not improve an individual’s perceived preparedness. The same three studies examined whether youth who received ILPs were more likely to have any financial accounts (*I*^2^ = 0.0%, *p* = .376; Figure 4, Panel M), a social security number (*I*^2^ = 0.0%, *p* = 0.756; Figure 4, Panel N), a birth certificate (*I*^2^ = 0.0%, *p* = .563; Figure 4, Panel O), or a driver’s license (*I*^2^ = 79.5%, *p* = .008; Figure 4, Panel P). All four outcomes were pooled using separate meta-analyses. The pooled SMD for all four outcomes—having a birth certificate (*g* = 0.24, 95% CI [−0.00, 0.48], *p* = .050), having any financial accounts (*g* = −0.01, 95% CI [−0.17, 0.16], *p* = .924), having a social security number (*g* = 0.04, 95% CI [−0.26, 0.34], *p* = .790), or having a driver’s license (*g* = 0.10, 95% CI [−0.29, 0.49], *p* = .622)—suggest that there was no difference on these measures between those who receive ILP and those that do not. The GRADE assessment established that confidence in all six meta-analyses was very low (⊕) due to being downgraded two levels for ROB, one level for imprecision and one level for inconsistency (driver’s license).

One RCT examined the impact of a TSP on individual job readiness (Table 9), for which no positive effect was observed (Leip, 2020).

**Table 9.** Life Skills Outcomes From Included Studies Not Included in a Meta-analysis.

Reference	Intervention	Outcome	Outcome Timing	*g* [95% CI]	Risk of Bias
Leip (2020)	True North	Job readiness index	8 months	0.07 [−0.16, 0.31]	High

## Discussion

There were limited primary studies of sufficient rigor to populate this review, almost all of which are characterized by substantial ROB. Of the 152 unique outcomes considered by this review, no detectible difference between the intervention and comparison was found in 98 (64.5%) of them. Where detectible differences were observed, effect sizes are mostly very small and only reported in individual studies—these did not persist when synthesized in a meta-analysis. The overall quality of the evidence in these studies is also very low. As a result, the scope and strength of current evidence is insufficient to draw firm conclusions about the effectiveness of any particular approach. One thing that can be said with a high level of certainty is that ILPs alone are insufficient to meaningfully change trajectories for care leavers. It may be that they are beneficial when combined with other services, but they appear to be insufficient on their own. These findings do not necessarily mean that this approach should be discarded entirely, but without considerable improvement and pairing with other approaches, outcomes for care leavers are unlikely to improve. That said, other than the opportunity cost of receiving an intervention that is unlikely to be effective, they did not appear to harm participants.

While this review has identified some emerging evidence that extended care can improve outcomes across a range of domains, we still have much to learn. Questions remain about the optimal delivery methods for such support: which specific groups of young people might need supplementary services? and what an ideal combination of additional support might look like?

### Implications of These Results

The critical findings of this review—summarized in Table 10—suggest that young people continue to leave care without the foundations and resources they need to thrive. The suite of policies and interventions analyzed here are not achieving their expected outcomes for care leavers. While individual interventions might provide some marginal improvements compared to services as usual or other programs of a similar nature, collectively, they are not sufficient to shift the needle for care leavers. It may be that we have an implementation problem, in that interventions are either delivered at the wrong time, with inadequate quality, or at an intensity or focus that is insufficient to address some of the complex challenges care leavers face. Extending care appears to be a positive step; however, it is not a panacea and will not alone solve all the complex challenges that care leavers face. The studies included in this review suggest that while it provides young people with additional time to prepare for independence, it appears to mostly delay, rather than prevent, negative outcomes (i.e., homelessness). Though delays of this nature can be of great benefit, and it is not yet clear whether early gains (i.e., university commencement) are sustained over time (i.e., degree completion). Although extended care policies in other jurisdictions may have aimed to address some of these issues, the limited available evidence prevents us from determining what constitutes a well-resourced and implemented extended care policy.

**Table 10.** Summary of Critical Findings From This Review.

• The evidence identified in this review is insufficient to recommend a particular policy or intervention. • Although some individual interventions could provide some marginal improvements compared to services as usual or other programs of a similar nature, collectively current policy and practice does not provide care leavers with the foundations and resources they need to thrive. • It is possible that interventions are either delivered at the wrong time, with inadequate quality, or at an insufficient intensity or focus. • Extending care may be a benefit. However, on its own, it is unlikely to solve the many complex challenges that care leavers face as it appears to mostly delay, rather than prevent, negative outcomes.

### Implications for Research

That this review contained 14 studies marks an improvement from the empty review conducted by Donkoh et al. (2006) over 17 years ago. While it is promising to see an increase in the number of studies, there continue to be few eligible studies within each high-level outcome, limiting the scope and power of any quantitative synthesis. There is considerable opportunity to both expand and strengthen research in this area. Reviews that have not applied methodological filters have identified a range of other interventions for care leavers that have not been rigorously evaluated (Everson-Hock et al., 2011; Heerde et al., 2018; Liu et al., 2019; O’Donnell et al., 2020; Randolph & Thompson, 2017). Moreover, given the cross-national interest in improving outcomes for care leavers, it is surprising that only few included studies were conducted outside the United States. Replication studies of promising interventions, particularly extended care, would be welcome, as would more experimental or quasi-experimental studies in countries outside the United States.

Future studies should consider the practice and policy contexts in which interventions are being delivered, taking into account the high degree of complexity of routine service settings affecting even the most well-designed interventions. There may be opportunities to test the feasibility and effectiveness of different implementation strategies to support the use of promising interventions or improve the delivery of existing services for example, to improve the implementation of services so that care leavers are able to access the support they are eligible for. This would allow us to better understand the difference that intentional implementation practice may have on client outcomes, through improving the delivery of either usual services or specific interventions. Given the relatively small effects observed in included interventions, along with scant information about implementation contained within these studies—notable exceptions being the Multisite Evaluation of Foster Youth Programs and YVLifeSet RCTs—it is plausible that efforts to measure and improve the implementation of these interventions could contribute to stronger effects and increased certainty (Hoagwood et al., 2020; Zanti & Thomas, 2021).

Many of the included interventions for care leavers provide similar components, e.g., case management, but since they are evaluated at the program level, we have little knowledge of which components may contribute to improved outcomes. Recognizing this, it would be helpful to identify and test which components of interventions drive improvements in outcomes for young people and are, therefore, important to nurture and maintain. A “common elements” approach—which identifies and leverages core components shared across various interventions with evidence of effectiveness—could be fruitful here (Chorpita et al., 2005; Garland et al., 2008). A necessary precondition for such research is that effectiveness studies clearly articulate the theory of change, the causal mechanisms, and the key elements of an intervention and the conditions required to implement it. A step further would be measuring the timing and dose of each element—including that provided in services as usual—which would allow future syntheses to assess their importance using sophisticated meta-analytic methods such as component network meta-analysis.

To facilitate direct comparisons of the impact of interventions for care leavers, it would also be beneficial if researchers collecting primary outcome data sought to use more common outcomes and measures. The technical reports of the Multi-site Evaluation of Foster Youth Programs (Courtney et al., 2008; Courtney, Zinn, Johnson et al., 2011; Courtney, Zinn, Koralek et al., 2011) and YVLifeSet (Valentine et al., 2015) offer some inspiration in this regard. However, translating these measures for use in settings outside the U.S. to thereby enable greater international collaboration in this field of research remains an important task.

### Implications for Policy and Practice

The transition from OOHC to independent living is a particularly challenging area of practice. Young people enter OOHC with complex trauma, receive varying quality of care, face challenges in finding a permanent place to stay, confront systematic inequality in access to the care they need from the wider system (e.g., psychological support, education, health, employment, etc.), and then need to be supported to live independently in both a relatively short period of time and at an age younger than their contemporaries who are not in OOHC. After leaving care, young people must navigate the structural barriers common to all young individuals, such as insecure employment and limited housing availability, but possess substantially less social and human capital than many, if not most, of their peers.

This review has some contributions for improving policy and practice in this area—see Table 11 for a summary. The current policy context in many high-income countries favors the expansion of extended care, which will likely continue. This is a positive development, as it is clear that young people are often not ready to leave care at age 18. Additionally, the policy is relatively easy to scale, and policymakers increasingly recognize the limitations of existing practice (Mendes et al., 2023; Mendes & Rogers, 2020; van Breda et al., 2020). The adoption of extended care should be seen as a prerequisite rather than an end-state, as it is unlikely to improve outcomes for care leavers when provided alone. The expansion of extended care should not displace the availability of additional support for care leavers. Not all care leavers have the same needs, and some are likely to require additional types of support (Mendes et al., 2023; Taylor et al., 2020).

**Table 11.** Summary of Implications for Practice and Policy From This Review.

• The expansion of extended care will likely continue as is relatively easy to scale and may have important short to medium-term benefit. However, it is unlikely to improve long-term outcomes for care leavers when provided alone. Additional support is likely needed. • Approaches commonly used in Implementation Science, such as implementation strategies or common elements/core components, could improve the delivery of some interventions. • The quality of “usual services” provided to care leavers could be improved using a continuous quality improvement approach.

Emerging adulthood is a period of change and uncertainty, yet our current approach to supporting care leavers assumes a linear progression from care to independence with limited or no opportunities to return after young people have left, or to access additional support. Furthermore, support for care leavers in high-income countries is generally provided in a uniform manner and prioritizes access to services and assistance rather than supporting the achievement of outcomes of importance to participants through the delivery of tailored support. The continued expansion of extended care provides an opportunity to reframe this approach by focusing on improving the quality of “usual services” provided to care leavers. At the policy level, this could involve targeted policy initiatives or systematic efforts by sector organizations and service agencies to change practice based on principles of continuous quality improvement (Zuchowski et al., 2019). At the practice level, this could involve trialing different implementation strategies or augmenting existing services with additional intervention elements and evaluating their impact. At both levels, such efforts would require resources, support and incentives for reviewing and enhancing current services, and decision makers in the field to truly operationalize and apply important service principles such as continuity and flexibility, autonomy and choice—but also accountability and responsibility (Meadowcroft et al., 2018).

### Limitations

For a review that sought to assess the literature at a global level, it is a limitation that all but one of our included studies was conducted in the United States. The health and social welfare “safety net” varies in scope and scale even within in the United States, and there is certainly variation between the United States and other high-income countries. This may affect the generalizability of some of the results reported in this review to other contexts.

### Diversity

Individuals from groups who have historically faced additional barriers, including ethnic minority and First Nations groups, are overrepresented among care leavers. Given the diversity in this population, the potential exists for policies and interventions to have heterogeneous effects by subgroups including gender, ethnic minority and First Nations status, and non-native speakers. Our review was unable to assess these differences as there were an insufficient number of included studies and/or there were limitations in the reporting of results. Future research should conduct and document subgroup analyses to explore outcome heterogeneity, an essential step for assessing the efficacy of policies and interventions across diverse and vulnerable populations.

## Conclusion

Young people leaving OOHC face elevated risks of harm, largely because they are often obliged to fend for themselves at an age when their counterparts in the community would still be able to rely on their families for financial and social support. The provision of appropriate and effective support to young people leaving OOHC, including extending care, is an issue of cross-national interest—particularly in high-income countries. Yet, despite this cross-national interest, almost all included studies that met our inclusion criteria were conducted in the United States. While some interventions, particularly extended care, show promise, the strength of existing evidence is insufficient to recommend any particular approach. Tinkering with existing interventions, particularly ILP, is unlikely to substantially improve outcomes for care leavers. Rather than focusing on specific interventions, there may be more opportunities to support care leavers by improving the implementation of “usual services” in many settings, including extended care which is the norm in many jurisdictions. As with extended care, we recommend that these improvement studies are evaluated using methods that allow the recovery of a causal estimand. If RCT are impractical, well-executed observational studies should be used.
